# The Association of Nutritional Risk Screening 2002 With 1-Year Re-hospitalization and the Length of Initial Hospital Stay in Patients With Heart Failure

**DOI:** 10.3389/fnut.2022.849034

**Published:** 2022-04-29

**Authors:** Zhezhe Chen, Hangpan Jiang, Wujian He, Duanbin Li, Maoning Lin, Min Wang, Min Shang, Wenbin Zhang

**Affiliations:** ^1^Department of Cardiology, College of Medicine, Sir Run Run Shaw Hospital, Zhejiang University, Hangzhou, China; ^2^Key Laboratory of Cardiovascular Intervention and Regenerative Medicine of Zhejiang Province, Hangzhou, China; ^3^Department of Cardiology, College of Medicine, The Fourth Affiliated Hospital, Zhejiang University, Yiwu, China

**Keywords:** heart failure, Nutritional Risk Screening 2002, 1-year re-hospitalization, length of initial hospital stay, nutritional risk

## Abstract

**Backgrounds and Aims:**

Nutritional Risk Screening 2002 (NRS-2002) has been widely recommended for identifying the nutritional risk. However, the association between NRS-2002 and the prognosis of heart failure has not been fully addressed. This study aimed to explore the association of NRS-2002 with 1-year re-hospitalization and the length of initial hospital stay in heart failure patients.

**Methods:**

This retrospective study included 2,830 heart failure patients. The primary endpoint was 1-year re-hospitalization for heart failure. The secondary endpoint was the length of initial hospital stay. The Log-binomial regression analysis was performed to determine the association between NRS-2002 and re-hospitalization. The Cox regression model was fitted to estimate hazard of discharge. The cumulative incidence curves of discharge were plotted using Kaplan–Meier method and log-rank test was performed. Exploratory analysis was also conducted according to the classification of heart failure and the level of N-terminal pro-B-type natriuretic peptide (NT-proBNP) fold-elevation.

**Results:**

Among 2,830 heart failure patients, the mean age was 64.3 years and 66.4% were male. A total of 122 (4.3%) patients were considered at high nutritional risk. Log-binomial regression analysis demonstrated that higher NRS-2002 score was an independent risk factor of re-hospitalization ([1 vs. 0]: relative risks [*RR*] = 1.383, 95% *CI* = 1.152 to 1.660; [2 vs. 0]: *RR* = 1.425, 95% *CI* = 1.108 to 1.832; [3–7 vs. 0]: *RR* = 1.770, 95% *CI* = 1.310 to 2.393). Kaplan–Meier curve showed that the cumulative incidence of discharge was lower in high nutritional risk group (Log rank *p* < 0.001). Cox regression analysis also found that higher NRS-2002 score (2 or ≥3) was strongly associated with longer length of initial hospital stay ([2 vs. 0]: Hazard ratios [*HR*] = 0.854, 95% *CI* = 0.748 to 0.976; [3–7 vs. 0]: *HR* = 0.609, 95% *CI* = 0.503 to 0.737). Exploratory analysis showed that such association still remained irrespective of NT-proBNP fold-elevation, but only existed in patients with heart failure with preserved ejection fraction (HFpEF).

**Conclusion:**

In patients with heart failure, high NRS-2002 score was strongly and independently associated with the incidence of 1-year re-hospitalization and the length of initial hospital stay.

## Introduction

Heart failure is a major and growing public health problem, which results in high mortality and re-hospitalization rate (1). Heart failure can be caused by any structural or functional cardiac disorder that impairs the ability of the ventricle to fill or eject blood, and is considered as the terminal stage of various cardiovascular diseases (2). The progression of heart failure is associated with a variety of risk factors, most notably inflammation status, coronary artery disease, hypertension, diabetes, and obesity, and they are tightly related to impaired nutritional status (3, 4). It is well-established that high nutritional risk is widespread among heart failure patients and turns to be one of the most important determinants in the length of hospital stay, in-hospital mortality, and cardiovascular events, especially in the elderly (5–7). Accordingly, nutritional risk assessment in early stages plays a vital role not only in preventing the deterioration of heart failure, but also in predicting the prognosis of heart failure (8).

For decades, several nutritional assessment tools have been proposed and extensively used in clinical settings, such as Nutritional Risk Screening 2002 (NRS-2002), Controlling Nutritional Status (CONUT), Geriatric Nutritional Risk Index (GNRI), Mini Nutritional Assessment (MNA) (9–11). Among them, NRS-2002 has emerged as a simple nutritional assessment tool, which was first developed by Kondrup et al. and is composed of patient's nutritional status, severity of diseases, and age (12). Unlike other nutritional assessment tools that mainly focus on laboratory indicators such as albumin, lymphocyte, and so on; NRS-2002 additionally takes the effect of the changes in food intake and disease severity into consideration. Nowadays, NRS-2002 stands out as an effective, flexible, and comprehensive nutritional assessment tool and has been extensively used in the clinical nutrition assessment to provide nutritional information (13). Studies have shown that NRS-2002 performed well in predicting prognosis especially the mortality and length of hospital stay among patients with different types of cancers (14), chronic kidney disease (15), and cardiovascular disease (16). Recent research also found that the high nutritional risk assessed by NRS-2002 was significantly associated with the long-term mortality in hospitalized patients with chronic heart failure (CHF) (17). However, whether NRS-2002 is associated with 1-year re-hospitalization for heart failure and the length of initial hospital stay in heart failure patients has not been fully understood.

Therefore, this observational study was conducted to investigate the association between NRS-2002 and the clinical outcomes in heart failure patients, such as 1-year re-hospitalization for heart failure and the length of initial hospital stay.

## Methods

### Study Population

In this observational study, 5,919 heart failure patients, who were hospitalized in Sir Run Run Shaw Hospital and its medical consortium hospitals from January 2009 to April 2019, were recruited. Patients with the following criteria were excluded: (1) NRS-2002 score was not evaluated or documented at admission; (2) missing data on baseline characteristics, laboratory measurements, or past medical history; (3) severe hepatic or renal dysfunction, active malignant tumor, or critical autoimmune disease; (4) pregnant or lactating women during hospitalization or follow-up. Finally, a total of 2,830 patients were enrolled. Strengthening the Reporting of Observational Studies in Epidemiology (STROBE) reporting standards were followed (18). Ethical approval was obtained from the ethics committee of Sir Run Run Shaw Hospital (NO.20201217-36).

### Sample Size Estimation

The estimated number of participants was based on the incidence of re-hospitalization in the previous study and on the principle of 10 outcome events per variable (19). A previous study reported that ~20% patients with heart failure would be re-admitted within 1-year (1). Therefore, 20% was adopted as the estimated incidence of re-hospitalization in patients with heart failure. According to the estimated incidence of re-hospitalization and nine variables included in the log-binomial regression model, at least 450 heart failure patients were needed, which was far less than actual enrollment.

### The Definitions of Heart Failure and NRS-2002

According to the 2016 European Society of Cardiology (ESC), heart failure is defined as an inability of the heart to meet the humans' metabolic demands, accompanied with typical symptoms and signs. The typical symptoms of heart failure mainly include dyspnea, weakness, chest tightness, and cardiogenic shock. The typical signs of heart failure involve lower limb edema, elevated jugular venous pressure, and pulmonary oedema (20). According to the level of left ventricular ejection fraction (LVEF), heart failure is classified into three types: heart failure with reduced ejection fraction (HFrEF) (<40%), heart failure with mid-range ejection fraction (HFmrEF) (40–49%), and heart failure with preserved ejection fraction (HFpEF) (≥50%).

Based on the European Society for Clinical Nutrition and Metabolism (ESPEN), the detailed calculation method of NRS-2002 was listed in Supplementary Table 1 (21). NRS-2002 score is calculated according to patients' impaired nutritional status, the severity of disease and age. The impaired nutritional status and severity of disease are scored between 0 and 3 points, respectively. Patients aged 70 years or older would receive an additional point based on the total score. Age-adjusted total NRS-2002 score ranges on a scale of 0 to 7. A total NRS-2002 score ≥3 points is considered at high nutritional risk, while those with a score below 3 points are considered at low nutritional risk (22).

### Study Endpoints

Patients who were first hospitalized for heart failure were enrolled in this study and were followed up for 1-year, regardless of the classification of heart failure. The primary endpoint was re-hospitalization for heart failure during a 1-year follow-up after discharge. And the secondary endpoint was the length of initial hospital stay, which was defined as the length of stay in patients who were hospitalized for heart failure for the first time and was calculated by subtracting the date of admission from the date of discharge.

### Data Collection

Baseline characteristics and clinical parameters related to this study were all derived from the Hospital Information System (HIS). Demographic data, laboratory data, comorbidities, and medication were collected. Fasting venous blood samples were collected on the morning after hospital admission day and then immediately sent to hematological and biochemical laboratory examinations. LVEF was assessed according to the classical Teichholz method. N-terminal pro-B-type natriuretic peptide (NT-proBNP) was analyzed using the NT-proBNP fold-elevation (actual value divided by the upper limit of normal value according to the age stratification). Nutritional status was assessed routinely within 24 h after hospital admission by the NRS-2002, which was applied by the trained nursing staff.

### Statistical Analysis

Continuous variables were presented as mean ± standard deviation (SD) if normally distributed, and median (interquartile range) if not. Categorical variables were expressed as numbers and proportions. Comparisons between high and low nutritional risk group were made using Student *t*-test if normally distributed or Mann–Whitney *U* test if not. The chi-square test or Fisher's exact test were used to examine the comparability of baseline characteristics for categorical variables according to minimal expected cell value.

The association between nutritional status (low nutritional risk and high nutritional risk) and 1-year re-hospitalization was evaluated using log-binomial regression models. Relative risks (RRs) with 95% *CI* were calculated. Covariates with potential significance for the prognosis of heart failure were adjusted in adjusted model 1, such as age (<65 or ≥65 years) (23), sex (male or female) (23), diabetes (yes or no) (24), hypertension (yes or no) (25), and estimated glomerular filtration rate (eGFR) (<90 or ≥90 ml/min/1.73 m^2^) (26). Adjusted model 2 further adjusted LVEF (<40, 40–49, or ≥50%) (27), NT-proBNP fold-elevation (<2 or ≥2) (28) and admission of diuretics (yes or no) (29). The association of NRS-2002 with the length of initial hospital stay was first visualized by loess smooth curve. Kaplan–Meier curve was generated to show the cumulative incidence of discharge in patients stratified by high and low nutritional risk and log-rank test was performed. Cox regression analysis was performed to estimate the hazard of discharge, with the same adjustment mentioned above. Hazard ratios (*HRs*) >1.0 represented shorter length of hospital stay, while *HRs* < 1.0 represented longer length of hospital stay. To further explore whether NRS-2002 remained effective in patients at low nutritional risk, the total population was divided into four categories (NRS-2002 score = 0, 1, 2, and ≥3). Log-binomial and Cox regression analysis were performed again and the NRS-2002 score = 0 group was regarded as the reference category. Finally, exploratory analysis was conducted according to the classification of heart failure and the level of NT-proBNP fold-elevation.

All statistical tests were 2-sided and *p*-value <0.05 was considered significant. All statistical analysis were performed with the Statistical Package for Social Science software version 25.0 (SPSS Inc., Chicago, USA) and R software version 4.0.5 (The R Foundation for Statistical Computing, Vienna, Austria).

## Results

### Patient Screening and Baseline Characteristics

**Figure 1:**
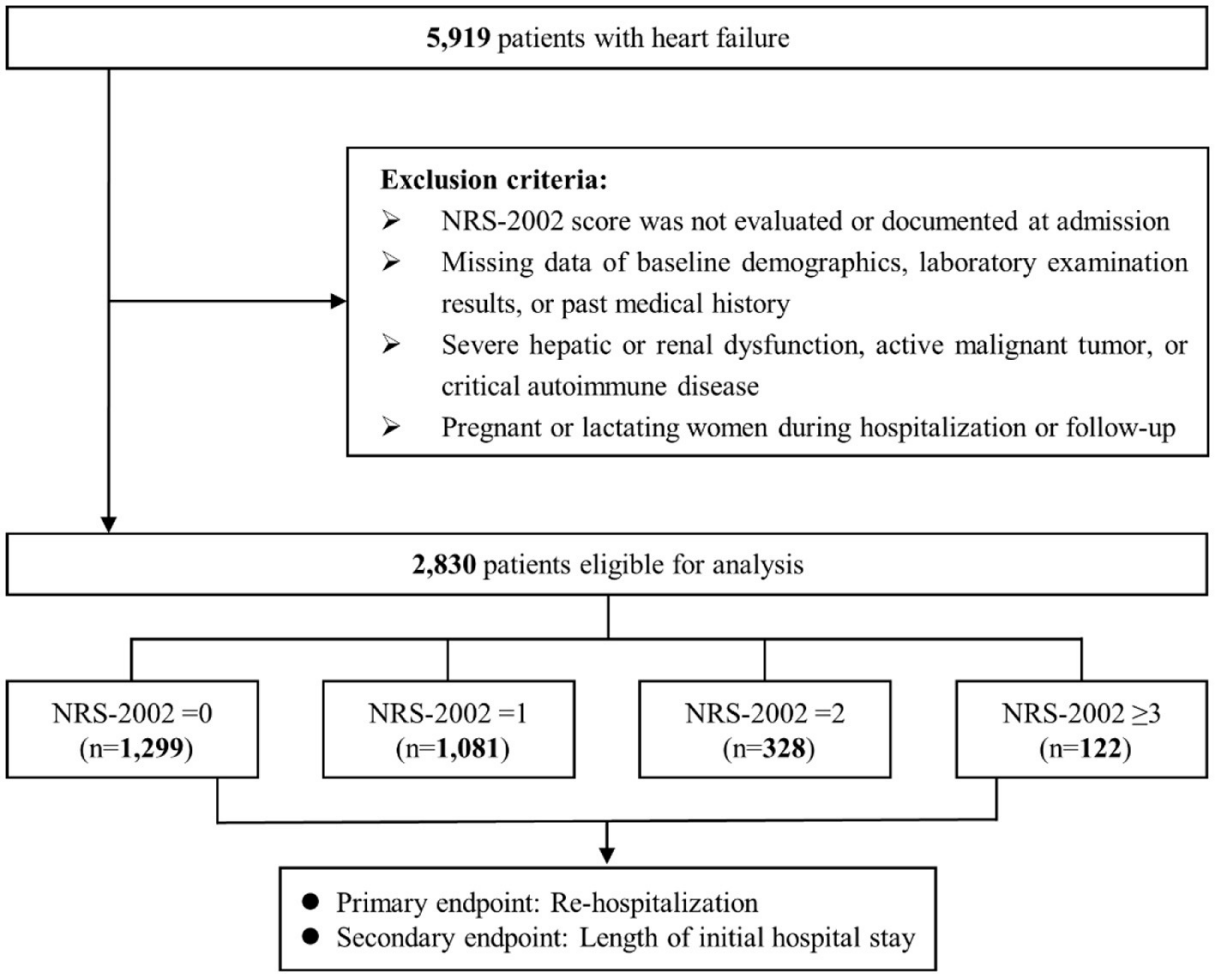
Flow diagram. NRS-2002 indicates Nutritional Risk Screening 2002.

As shown in Figure 1, a total of 5,919 patients with heart failure were screened for eligibility and 2,830 patients were finally enrolled. Table 1 presents the baseline demographics and clinical features of enrolled patients. The average age was 64.3 ± 11.9 years and 1,880 (66.4%) were male. A total of 122 (4.3%) patients were at high nutritional risk (NRS-2002 score ≥3). A total of 568 (20.1%) patients were re-hospitalized for heart failure after discharge in 1-year follow-up. The median length of initial hospital stay was 5.0 [3.0, 8.0] days. Compared with low nutritional risk group, patients at high nutritional risk had a higher incidence of re-hospitalization (30.3 vs. 19.6%, *p* = 0.006), and longer length of initial hospital stay (7.0 [5.3, 12.0] days vs. 5.0 [3.0, 7.0] days, *p* < 0.001). Moreover, high nutritional risk patients had worse cardiac function with lower LVEF (54.5 [41.1, 60.0]% vs. 57.7 [43.5, 65.0]%, *p* = 0.016) and higher NT-proBNP fold-elevation (2.23 [1.44, 4.35] vs. 1.86 [1.23, 3.08], *p* = 0.005), and were more likely to be treated with diuretics (39.3 *vs*. 22.9%, *p* < 0.001) and nitroglycerin (68.9 vs. 49.4%, *p* < 0.001). However, there was no statistical difference in sex, smoking status, eGFR, total cholesterol (TC), low density lipoprotein cholesterol (LDL-C), presence of diabetes, hypertension and ischemic cardiomyopathy, and medication with milrinone and cedi-lanid (all *p*-values >0.05).

**Table 1:** Baseline characteristics of patients stratified by nutritional status.

		**Nutrition Risk Screening-2002**			
		**Overall (*n* = 2,830)**	** <3 (*n* = 2,708)**	**≥3 (*n* = 122)**	***P*-value**
**Demographic features**
	Age, years old	64.3 ± 11.9	64.2 ± 11.8	67.7 ± 13.2	0.002*
	Male, *n* (%)	1,880 (66.4)	1,797 (66.4)	83 (68.0)	0.776
	BMI, kg/m^2^	24.1 ± 3.3	24.1 ± 3.3	23.1 ± 3.9	0.004*
	LVEF, %	57.5 [43.3, 65.0]	57.7 [43.5, 65.0]	54.5 [41.1, 60.0]	0.016*
	Current smoking, *n* (%)	988 (34.9)	950 (35.1)	38 (31.1)	0.427
	Re-hospitalization, *n* (%)	568 (20.1)	531 (19.6)	37 (30.3)	0.006*
	Length of in-hospital stay, days	5.0 [3.0, 8.0]	5.0 [3.0, 7.0]	7.0 [5.3, 12.0]	<0.001*
**Laboratory data**
	eGFR, ml/(min × 1.73 m^2^)	86.0 [74.3, 95.1]	86.0 [74.4, 95.1]	84.2 [71.6, 94.2]	0.235
	TC, mmol/L	4.02 ± 1.00	4.02 ± 1.00	3.96 ± 1.04	0.578
	LDL-C, mmol/L	2.21 ± 0.76	2.21 ± 0.76	2.19 ± 0.77	0.796
	Triglyceride, mmol/L	1.45 ± 0.91	1.46 ± 0.92	1.26 ± 0.68	0.027*
	NT-proBNP, pg/ml	1791.0 [1087.0, 3018.5]	1774.0 [1082.8, 2945.5]	2339.0 [1301.5, 4129.3]	<0.001*
	NT-proBNP fold-elevation	1.88 [1.23, 3.14]	1.86 [1.23, 3.08]	2.23 [1.44, 4.35]	0.005*
	CRP, mg/L	2.3 [0.9, 7.8]	2.3 [0.9, 7.5]	3.7 [1.3, 16.2]	0.001*
	Albumin, g/L	39.2 ± 4.5	39.3 ± 4.4	37.2 ± 5.5	<0.001*
**Comorbidities**, ***n*** **(%)**
	Diabetes	566 (20.0)	540 (19.9)	26 (21.3)	0.799
	Hypertension	1,521 (53.7)	1,449 (53.5)	72 (59.0)	0.271
	Coronary heart disease	1,161 (41.0)	1,095 (40.4)	66 (54.1)	0.004*
	Ischemic cardiomyopathy	86 (3.0)	85 (3.1)	1 (0.8)	0.234
**Medication**, ***n*** **(%)**
	Diuretics	667 (23.6)	619 (22.9)	48 (39.3)	<0.001*
	Nitroglycerin	1,422 (50.2)	1,338 (49.4)	84 (68.9)	<0.001*
	Milrinone	125 (4.4)	119 (4.4)	6 (4.9)	0.960
	Cedi-lanid	152 (5.4)	146 (5.4)	6 (4.9)	0.983

Data are expressed as mean ± standard deviation or median (interquartile range) in continuous variables according to distribution, and numbers (proportions) in categorical variables. NRS-2002, Nutritional Risk Screening 2002; BMI, body mass index; LVEF, left ventricular ejection fraction; TC, total cholesterol; LDL-C, low density lipoprotein cholesterol; CRP, C-reactive protein; eGFR, estimated glomerular filtration rate; NT-proBNP, N-terminal pro-B-type natriuretic peptide. ^*^P <0.05.

### Association of NRS-2002 With 1-Year Re-hospitalization

**Figure 2:**
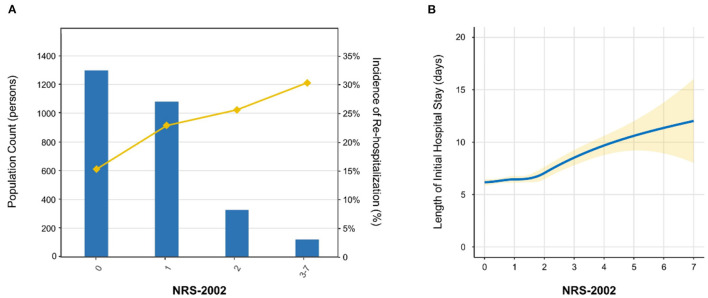
Population distribution and loess smooth curve. **(A)** The distribution of study population and the incidence of re-hospitalization. The histogram showed the population distribution of study subjects stratified by NRS-2002 categories, while the line chart showed the trend of incidence of re-hospitalization. **(B)** Loess smooth curve of NRS-2002 score with the length of initial hospital stay. The loess smooth curve was fitted for exploring the association between NRS-2002 score and the length of initial hospital stay. The yellow shadow around the solid line represents 95% confidence interval. NRS-2002 indicates Nutritional Risk Screening 2002.

Figure 2A shows the population distribution stratified by nutritional categories (NRS-2002 score = 0, 1, 2, and ≥3), and the increasing trend of the incidence of re-hospitalization with the increase of NRS-2002 score. Log-binomial regression analysis was performed to reveal the association of nutritional risk assessed by NRS-2002 with 1-year re-hospitalization. Supplementary Table 2 shows that high nutritional risk (NRS-2002 score ≥3 vs. <3) was an independent risk factor of re-hospitalization (Adjusted model 2: *RR* = 1.424, 95% confidence interval (*CI*) = 1.083 to 1.871). Then, NRS-2002 was classified into four categories and the NRS-2002 = 0 group was considered as reference category. Higher NRS-2002 score was still found to be strongly and independently associated with higher incidence of re-hospitalization (Adjusted model 2: [1 vs. 0]: *RR* = 1.383, 95% confidence interval (*CI*) = 1.152 to 1.660; [2 vs. 0]: *RR* = 1.425, 95% *CI* = 1.108 to 1.832; [3–7 vs. 0]: *RR* = 1.770, 95% *CI* = 1.310 to 2.393) (Table 2).

**Table 2:** Log-binomial regression analysis of NRS-2002 categories with 1-year re-hospitalization.

**NRS-2002**	**Unadjusted model**		**Adjusted model 1**		**Adjusted model 2**	
	**RR [95% CI]**	***P*-value**	**RR [95% CI]**	***P*-value**	**RR [95% CI]**	***P*-value**
0	1 (Reference)		1 (Reference)		1 (Reference)	
1	1.498 [1.266 to 1.772]	<0.001*	1.388 [1.156 to 1.666]	<0.001*	1.383 [1.152 to 1.660]	<0.001*
2	1.672 [1.336 to 2.092]	<0.001*	1.431 [1.111 to 1.841]	0.005*	1.425 [1.108 to 1.832]	0.006*
3–7	1.980 [1.470 to 2.666]	<0.001*	1.801 [1.334 to 2.432]	<0.001*	1.770 [1.310 to 2.393]	<0.001*

*NRS-2002 score = 0 group was set as reference category*.

*Unadjusted model adjusted for none*.

*Adjusted model 1 adjusted for age (<65 or ≥65 years), sex (male or female), diabetes (yea or no), hypertension (yes or no) and eGFR (<90 or ≥90 ml/min/1.73 m^2^)*.

*Adjusted model 2 additionally adjusted for LVEF (<40, 40–49, or ≥50%), NT-proBNP fold-elevation (<2 or ≥2) and admission of diuretics (yes or no)*.

NRS-2002, Nutritional Risk Screening 2002; RR, relative risk; CI, confidence interval; eGFR, estimated glomerular filtration rate; LVEF, left ventricular ejection fraction; NT-proBNP, N-terminal pro-B-type natriuretic peptide. ^*^P <0.05.

### Association of NRS-2002 With the Length of Initial Hospital Stay

**Figure 3:**
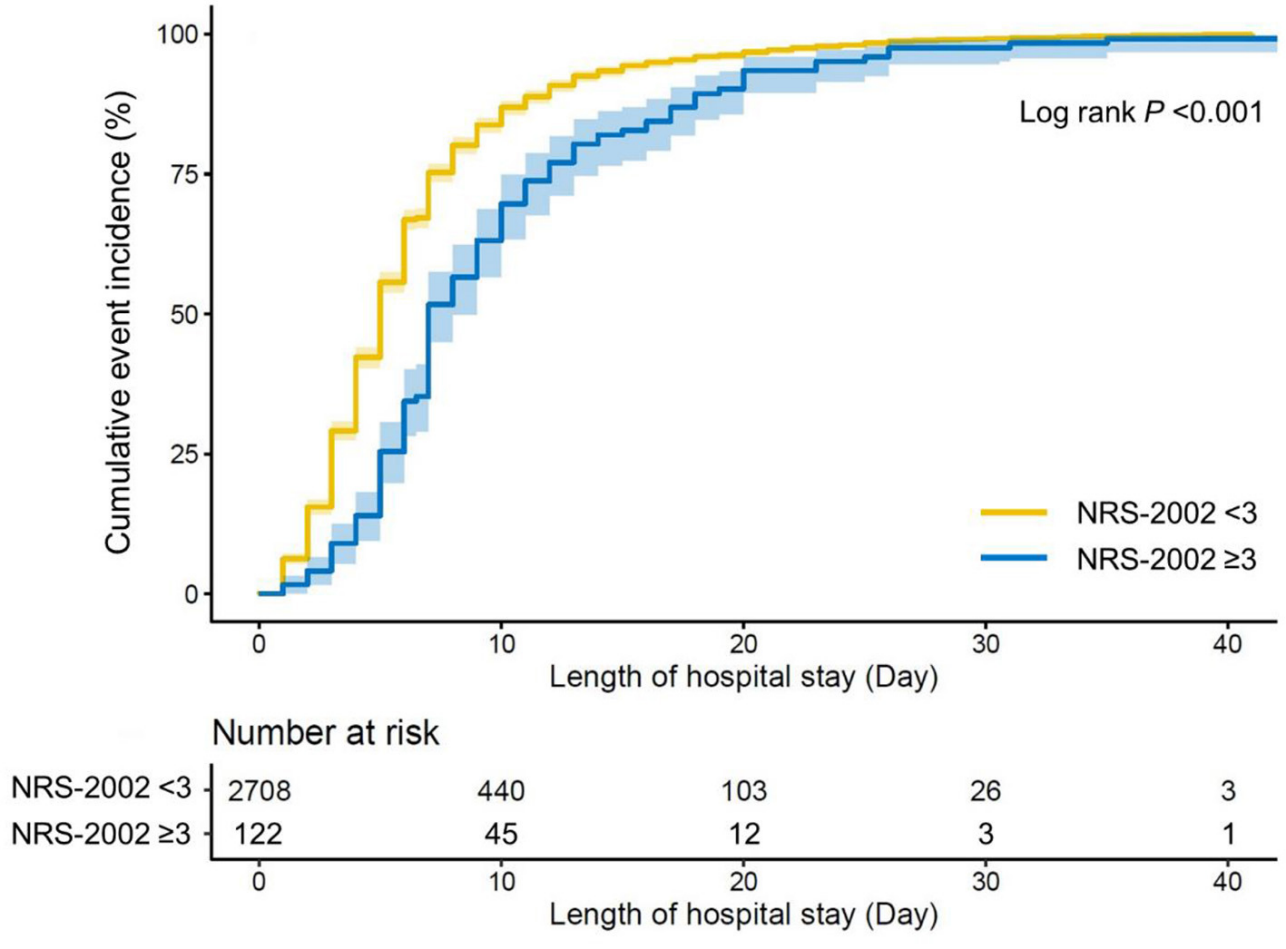
Cumulative incidence of discharge stratified by nutritional status. Kaplan–Meier curve was plotted to show the cumulative incidence of discharge in patients with low nutritional risk and high nutritional risk. Log rank *p*-value was also shown. NRS-2002 indicates Nutritional Risk Screening 2002.

Loess smooth curve was plotted, showing that with the increase of NRS-2002 score, the length of initial hospital stay increased correspondingly (Figure 2B). The cumulative incidence of discharge was also lower in the high nutritional risk group (Log rank *p* < 0.001) (Figure 3). Cox regression models verified that a high nutritional risk (NRS-2002 score ≥3 vs. <3) was associated with the increasing length of initial hospital stay (Adjusted model 2: *HR* = 0.636, 95% *CI* = 0.529 to 0.764) (Supplementary Table 2). Then, NRS-2002 was classified into four categories and the NRS-2002 = 0 group was regarded as a reference category. It was found that higher NRS-2002 score (2 or ≥3) was still tightly and independently associated with longer length of initial hospital stay (Adjusted model 2: [2 vs. 0]: *HR* = 0.854, 95% *CI* = 0.748 to 0.976; [3–7 vs. 0]: *HR* = 0.609, 95% *CI* = 0.503 to 0.737) (Table 3).

**Table 3:** Cox regression analysis of NRS-2002 categories with time to discharge.

**NRS-2002**	**Unadjusted model**		**Adjusted model 1**		**Adjusted model 2**	
	**HR [95% CI]**	***P*-value**	**HR [95% CI]**	***P*-value**	**HR [95% CI]**	***P*-value**
0	1 (Reference)		1 (Reference)		1 (Reference)	
1	0.950 [0.877 to 1.030]	0.215	0.936 [0.856 to 1.024]	0.151	0.959 [0.876 to 1.049]	0.360
2	0.854 [0.757 to 0.965]	0.011*	0.851 [0.745 to 0.972]	0.017*	0.854 [0.748 to 0.976]	0.020*
3–7	0.584 [0.484 to 0.704]	<0.001*	0.566 [0.468 to 0.685]	<0.001*	0.609 [0.503 to 0.737]	<0.001*

*NRS-2002 score = 0 group was set as reference category*.

*Unadjusted model adjusted for none*.

*Adjusted model 1 adjusted for age (<65 or ≥65 years), sex (male or female), diabetes (yea or no), hypertension (yes or no) and eGFR (<90 or ≥90 ml/min/1.73 m^2^)*.

*Adjusted model 2 additionally adjusted for LVEF (<40, 40–49, or ≥50%), NT-proBNP fold-elevation (<2 or ≥2) and admission of diuretics (yes or no)*.

NRS-2002, Nutritional Risk Screening 2002; HR, hazard ratio; CI, confidence interval; eGFR, estimated glomerular filtration rate; LVEF, left ventricular ejection fraction; NT-proBNP, N-terminal pro-B-type natriuretic peptide. ^*^P <0.05.

### Exploratory Analysis

**Figure 4:**
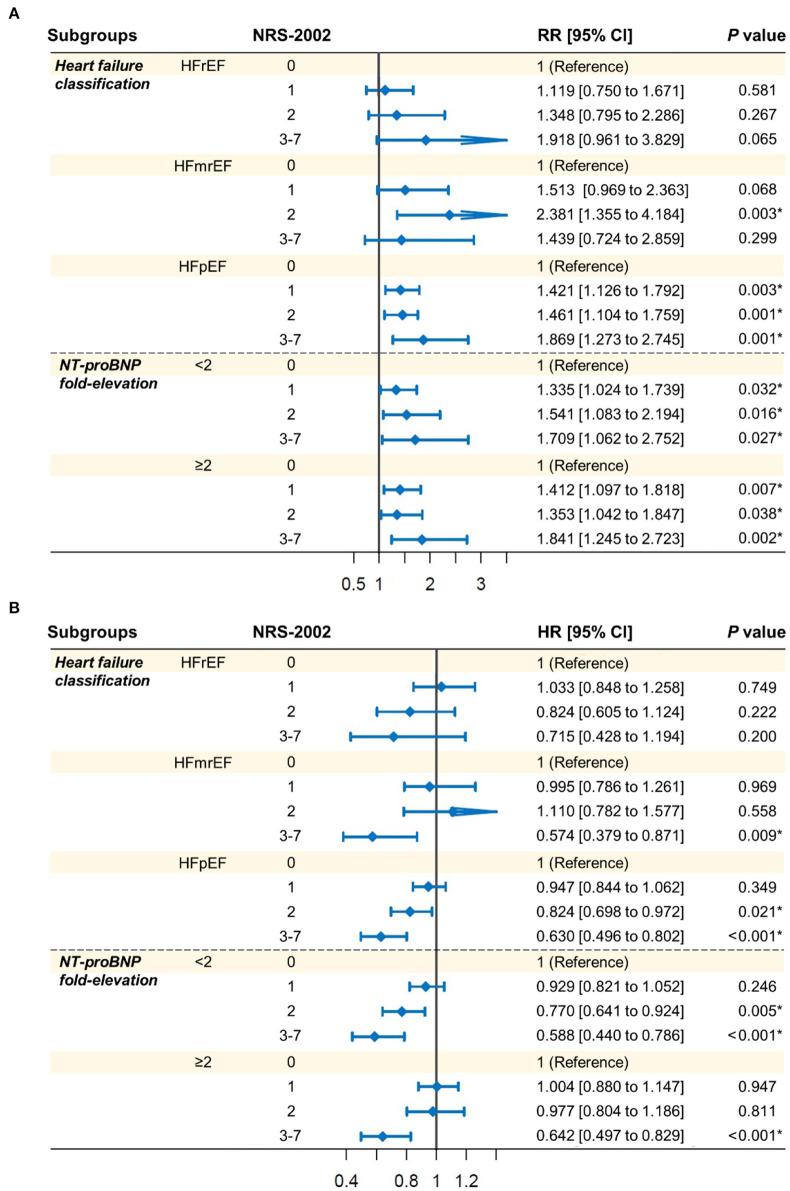
Exploratory analysis in the association of NRS-2002 categories with **(A)** 1-year re-hospitalization and **(B)** the length of initial hospital stay. Patients were classified according to heart failure classification (HFrEF, EFmrEF, or HFpEF) and the level of NT-proBNP fold-elevation (<2 or ≥2). Log-binomial regression analysis **(A)** and Cox regression analysis **(B)** were performed for different endpoints with the same covariates as in adjusted model 2 in Table 2, respectively. NRS-2002, Nutritional Risk Screening 2002; RR, relative risk; HR, hazard ratio; CI, confidence interval; HFrEF, heart failure with reduced ejection fraction; HFmrEF, heart failure with mid-range ejection fraction; HFpEF, heart failure with preserved ejection fraction; NT-proBNP, N-terminal pro-B-type natriuretic peptide.

To further explore the effect of NRS-2002 in different subgroups, an exploratory analysis was carried out according to heart failure classification (HFrEF, HFmrEF, or HFpEF) and the level of NT-proBNP fold-elevation (<2 or ≥2). Results of the association between NRS-2002 categories and 1-year re-hospitalization or the length of initial hospital stay were presented in Figure 4, respectively. Figure 4A shows that the positive association of a high NRS-2002 score with 1-year re-hospitalization still remained regardless of NT-proBNP fold-elevation level, but remained only in patients with HFpEF (all *p*-values <0.05). The results in Figure 4B showed that a high NRS-2002 score (NRS-2002 = 2 or ≥3) was tightly associated with a longer length of initial hospital stay in HFpEF patients or patients whose NT-proBNP fold-elevation <2, while in patients whose NT-proBNP more than 2, such positive association could only be observed when NRS-2002 ≥3.

## Discussion

In this observational study, nutritional risk assessed by NRS-2002 at admission was strongly and independently associated with re-hospitalization and the length of initial hospital stay in patients with heart failure. Specifically, high NRS-2002 score was an independent risk factor for 1-year re-hospitalization and the length of initial hospital stay. More importantly, exploratory analysis indicated that such association of NRS-2002 with re-hospitalization or the length of initial hospital stay generally existed irrespective of NT-proBNP fold-elevation, but only remained in patients with HFpEF.

Poor nutritional status and heart failure have a close association and commonly co-occur (30). Impaired nutritional status has been considered as one of the most critical risk factors of poor clinical outcomes in heart failure patients, especially in the elderly patients (6, 31). Therefore, it is of great interest to early evaluate the nutritional status of heart failure patients. A variety of nutritional assessment tools have been proposed and used in issued studies (32). Honda et al. and Joaquín et al. reported that both GNRI and MNA were useful tools to identify the nutritional risk in heart failure patients (33, 34). Kato et al. found that a high CONUT score was associated with a higher risk for in-hospital mortality and infection in patients with acute heart failure (AHF) (35). NRS-2002 was one of the accurate and flexible tools for nutritional status assessment, recommended by the Global Leadership Initiative on Malnutrition (GLIM) (36). Therefore, the current study chose NRS-2002 to assess heart failure patients' nutritional risk status and demonstrated that a high NRS-2002 score was a significant predictor of re-hospitalization and the length of initial hospital stay for patients with heart failure.

As a simple and convenient tool, NRS-2002 aims to identify nutritional at-risk individuals so that extra intervention can be initiated before the signs of malnutrition become obvious (37). The potential pathological mechanisms between NRS-2002 and the prognosis of heart failure are complex. All of the components of NRS-2002 can lead to an imbalance in energy synthesis and breakdown, which further induces oxidative stress, causes myocardial cell injury and ultimately leads to adverse cardiovascular events (38–41). Tevik et al. reported that NRS-2002 was significantly related to long-term mortality in hospitalized patients with CHF (16). Going further, the current study demonstrated that NRS-2002 was not only associated with the incidence of 1-year re-hospitalization, but also with the length of initial hospital stay. Therefore, it was crucial for heart failure patients' management and prognosis to early assess their nutritional status by NRS-2002.

Clinically, heart failure is a complex syndrome and is classified into three major types based on LVEF: HFrEF, HFmrEF, and HFpEF (20). HFpEF has become the major form of heart failure and researchers have found that the prognosis of HFpEF was not better than HFrEF patients, deserving more attention (42). To this end, the current study incorporated all types of heart failure populations involving HFrEF, HFmrEF, and HFpEF to better determine the effect of NRS-2002 in different types of heart failure. The results in Figure 4 showed that a high NRS-2002 score had a significant association with the incidence of re-hospitalization and the length of initial hospital stay only in HFpEF patients. It might indicate that there existed a limitation of NRS-2002 in predicting the re-hospitalization and the length of initial hospital stay in HFrEF and HFmrEF patients.

It was reported that NT-proBNP was a strong predictor of outcomes in heart failure patients (43). To eliminate the effects of NT-proBNP, in this study, log-binomial regression analysis and Cox regression analysis were conducted after the adjustment for covariates involving NT-proBNP fold-elevation. Besides, the exploratory analysis found that the positive association of high NRS-2002 score with re-hospitalization and the length of initial hospital stay still remained significant in heart failure patients with different NT-proBNP fold-elevation levels (<2 or ≥2), suggesting the validity of NRS-2002 in clinical practice.

The proportion of the high nutritional risk population among patients with heart failure differed significantly in previous studies (4.23–57%) (44–46). In the present study, the proportion of the high nutritional risk population (about 4.31%) was relatively low. In Tevik's research, only patients with LVEF ≤ 50% were included, which might result in the proportion of the high nutritional risk population higher (up to 57%) (44). The current study included patients with all types of heart failure, and the proportion of HFpEF was 66.8%. The nutritional status of HFpEF patients was relatively better than HFrEF patients, resulting in a lower proportion of high nutritional risk in the overall population of this study. The study by Czapla et al. also included patients with HFrEF, HFmrEF, and HFpEF, and the proportion of high nutritional risk (about 4.23%) was close to the proportion in this study (45).

This study also had some limitations that needed to be noticed. First, as a retrospective and observational study, the selection bias was unavoidable. Therefore, large prospective research should be conducted to support our findings. Second, in clinical practice, some patients at severe nutritional risk might receive relevant intervention by physicians or nutritionists during hospitalization or follow-up, such as nutritional supplementation, which might influence our results. Third, nutritional intake during hospitalization and follow-up, which is highly associated with not only nutritional status but also heart failure prognosis, was not available and needed to be considered as an important confounder in further research. Fourth, owing to the lack of the gold standard tool for nutritional assessment, the current study only investigated the role of NRS-2002 in the prognosis of patients with heart failure. Further studies need to focus on other nutritional assessment tools and compare the predictive performance in different tools.

## Conclusion

High NRS-2002 score was strongly and independently associated with the incidence of 1-year re-hospitalization and the length of initial hospital stay in heart failure patients.

## Data Availability Statement

The original contributions presented in the study are included in the article/Supplementary Materials, further inquiries can be directed to the corresponding author/s.

## Ethics Statement

The studies involving human participants were reviewed and approved by Ethics Committee of Sir Run Run Shaw Hospital of Zhejiang University (20200803-34). Written informed consent for participation was not required for this study in accordance with the national legislation and the institutional requirements.

## Author Contributions

WZ and MS conceived and designed the study. ZC organized these data and drafted the manuscript with the help of HJ, WH, DL, ML, and MW. ZC and HJ analyzed the data. WH drew the pictures. WZ, MS, and MW detected any errors in the whole process. All authors have read and approved the manuscript for submission.

## Funding

This work was supported by grants from the National Natural Science Foundation of China (82070408), the Medical Health Science and Technology Project of Zhejiang Provincial Health Commission (2021RC014), and the Traditional Chinese Medicine Science and Technology Project of Zhejiang Province (2021ZB172).

## Conflict of Interest

The authors declare that the research was conducted in the absence of any commercial or financial relationships that could be construed as a potential conflict of interest.

## Publisher's Note

All claims expressed in this article are solely those of the authors and do not necessarily represent those of their affiliated organizations, or those of the publisher, the editors and the reviewers. Any product that may be evaluated in this article, or claim that may be made by its manufacturer, is not guaranteed or endorsed by the publisher.

## Abbreviations

NRS-2002: nutritional risk screening-2002
CONUT: controlling nutritional status
GNRI: geriatric nutritional risk index
MNA: mini nutritional assessment
CHF: chronic heart failure
STROBE: Strengthening the Reporting of Observational Studies in Epidemiology
ESC: European Society of Cardiology
LVEF: left ventricular ejection fraction
HFrEF: heart failure with reduced ejection fraction
HFmrEF: heart failure with mid-range ejection fraction
HFpEF: heart failure with preserved ejection fraction
ESPEN: European Society for Clinical Nutrition and Metabolism
HIS: Hospital Information System
SD: standard deviation
RRs: relative risks
HRs: hazard ratios
CI: confidence interval
eGFR: estimated glomerular filtration rate
NT-proBNP: N-terminal pro-B-type natriuretic peptide
TC: total cholesterol
LDL-C: low density lipoprotein cholesterol
AHF: acute heart failure
GLIM: Global Leadership Initiative on Malnutrition.
