# Supplementary material for: The Association of Nutritional Risk Screening 2002 With 1-Year Re-hospitalization and the Length of Initial Hospital Stay in Patients With Heart Failure
Source: Front Nutr. 2022 Apr 29;9:849034. doi: 10.3389/fnut.2022.849034 (PMC9103872; doi:10.3389/fnut.2022.849034)
Supplement: Supplementary file 1 [file Table_1.docx]

Table S1 Nutritional Risk Screening 2002 (NRS-2002).

| **Nutritional Risk Screening 2002** | | | | | |
| --- | --- | --- | --- | --- | --- |
| **Impaired Nutritional Status** | | | **Score** | |  |
|  | 0 | Normal nutritional status. |  |  |  |
|  | 1 | Weigh loss >5% in 3 months, or food intake 50-75% of normal requirement in preceding week. |  |  |  |
|  | 2 | Weigh loss >5% in 2 months, or BMI 18.5-20.5kg/m^2^ + impaired general condition, or food intake 25-50% of normal requirement in preceding week. |  |  |  |
|  | 3 | Weigh loss >5% in 1 months (>15% in 3 months), or BMI <18.5 kg/m^2^ + impaired general condition, or food intake 0-25% of normal requirement in preceding week. |  |  |  |
| **Severity of Disease** | | | **Score** | |  |
|  | 0 | Normal nutritional requirements. |  |  |  |
|  | 1 | Hip fracture; Chronic diseases, in particular with acute complications: cirrhosis, COPD, hemodialysis; diabetes; oncology. |  |  |  |
|  | 2 | Major abdominal surgery; Stroke; Severe pneumonia; Hematologic malignancy. |  |  |  |
|  | 3 | Craniocerebral injury; Bone marrow transplantation; Intensive care patients (APACHE >10). |  |  |  |
| **Age** | | | **Score** | |  |
|  | 0 | <70 years |  |  |  |
|  | 1 | ≥70 years |  |  |  |
| **Total Score** | | |  | |  |

A total score of NRS-2002 ranges from 0 to 7. NRS-2002 <3: patients at low nutritional risk; NRS-2002 ≥3: patients at high nutritional risk.

BMI indicates body mass index; COPD, chronic obstructive pulmonary diseases; APACHE, Acute Physiology and Chronic Health Evaluation.
