# Supplementary material for: The Association of Nutritional Risk Screening 2002 With 1-Year Re-hospitalization and the Length of Initial Hospital Stay in Patients With Heart Failure
Source: Front Nutr. 2022 Apr 29;9:849034. doi: 10.3389/fnut.2022.849034 (PMC9103872; doi:10.3389/fnut.2022.849034)
Supplement: Supplementary file 2 [file Table_2.docx]

Table S2 Association between NRS-2002 (<3 or ≥3) and one-year re-hospitalization or time to discharge.

|  | NRS-2002 | Unadjusted Model |  |  | Adjusted model 1 |  |  | Adjusted model 2 |  |
| --- | --- | --- | --- | --- | --- | --- | --- | --- | --- |
|  |  | RR or HR [95% CI] | *P* value |  | RR or HR [95% CI] | *P* value |  | RR or HR [95% CI] | *P* value |
| Primary endpoint | <3 | 1 (Reference) |  |  | 1 (Reference) |  |  | 1 (Reference) |  |
|  | ≥3 | 1.547 [1.169 to 2.046] | 0.002* |  | 1.445 [1.101 to 1.898] | 0.008* |  | 1.424 [1.083 to 1.871] | 0.011* |
| Secondary endpoint | <3 | 1 (Reference) |  |  | 1 (Reference) |  |  | 1 (Reference) |  |
|  | ≥3 | 0.608 [0.507 to 0.730] | <0.001* |  | 0.597 [0.497 to 0.717] | <0.001* |  | 0.636 [0.529 to 0.764] | <0.001* |

NRS-2002 score <3 group was set as reference category.

Unadjusted model adjusted for none.

Adjusted model 1 adjusted for age (<65 or ≥65 years), sex (male or female), diabetes (yea or no), hypertension (yes or no) and eGFR (<90 or ≥90ml/min/1.73 m^2^).

Adjusted model 2 additionally adjusted for LVEF (<40, 40-49 or ≥50%), NT-proBNP fold-elevation (<2 or ≥2) and admission of diuretics (yes or no).

NRS-2002 indicates Nutritional Risk Screening 2002; RR, relative risk; HR, hazard ratio; CI, confidence interval; eGFR, estimated glomerular filtration rate; LVEF, left ventricular ejection fraction; NT-proBNP, N-terminal pro-B-type natriuretic peptide. **P* <0.05.
